# Catheter ablation for atrial fibrillation in patients with tricuspid regurgitation

**DOI:** 10.1016/j.hroo.2025.05.015

**Published:** 2025-05-17

**Authors:** Corinne Isenegger, Jonas Brügger, Gianmarco M. Balestra, Beat A. Kaufmann, Fabian Jordan, Sven Knecht, Philipp Krisai, David Spreen, Nicolas Schaerli, Felix Mahfoud, Christian Sticherling, Michael Kühne, Patrick Badertscher

**Affiliations:** 1 Department of Cardiology, University Hospital Basel, University of Basel, Basel, Switzerland; 2 Cardiovascular Research Institute Basel, University Hospital Basel, University of Basel, Basel, Switzerland

**Keywords:** Atrial fibrillation, Catheter ablation, Echocardiography, Pulmonary vein isolation, Tricuspid regurgitation, Valvular heart disease

**Key Findings**

▪ In patients with atrial fibrillation and moderate or severe tricuspid regurgitation (TR), catheter ablation (CA) was associated with a 1-year arrhythmia-free survival rate of 60%.
▪ Improvement in TR severity by at least 1 grade was observed in 63% of patients following CA, with significantly greater improvement among those without arrhythmia recurrence.
▪ All ablation modalities, including pulsed-field ablation, were feasible in this population, with no significant difference in outcomes between energy sources.
▪ These findings suggest that successful rhythm control via CA may contribute to reverse remodeling and improvement in TR, highlighting the importance of early rhythm intervention in this patient group.

Contemporary guidelines recommend early rhythm control strategies, including catheter ablation (CA) for patients with symptomatic atrial fibrillation (AF). However, the impact of coexisting tricuspid regurgitation (TR) on procedural characteristics and the efficacy of CA for AF remains unclear.

This retrospective analysis of patients with moderate or severe TR undergoing CA for AF in the Swiss Atrial Fibrillation Pulmonary Vein Isolation Registry. All patients underwent transesophageal echocardiography 1 day before CA. Patient characteristics, procedural details, and long-term follow-up data, including serial echocardiograms (ECGs), outpatient clinic visits, and Holter-ECG were collected for all patients. This study aimed to assess procedural characteristics and the efficacy of CA in patients with AF and moderate or severe TR. The study was approved by the local ethics committees and adhered to the Declaration of Helsinki. All patients provided written informed consent.

A total of 133 patients were included, with 127 patients (95%) having moderate TR and 6 patients (5%) having severe TR. The median age was 72 (68–76) years, and 55% were female. Paroxysmal AF was present in 57 patients (43%), while 76 patients (57%) had persistent AF. Left atrial diameter, left ventricular ejection fraction, right ventricular/right atrial gradient, pulmonary artery systolic pressure, and right atrial area were 43 mm (39–48 mm), 56% (45%–63%), 27 mm Hg (21–35 mm Hg), 35 mm Hg (29–42 mm Hg), and 25 cm^2^ (20–32 cm^2^), respectively. At baseline, 65 patients (49%) were in AF, 7 (5%) in atrial flutter and 61 (46%) in sinus rhythm. The ablation modalities used were radiofrequency in 51% of patients, pulsed-field ablation in 38% of patients, and cryoballoon ablation in 11% of patients.

**Figure 1:**
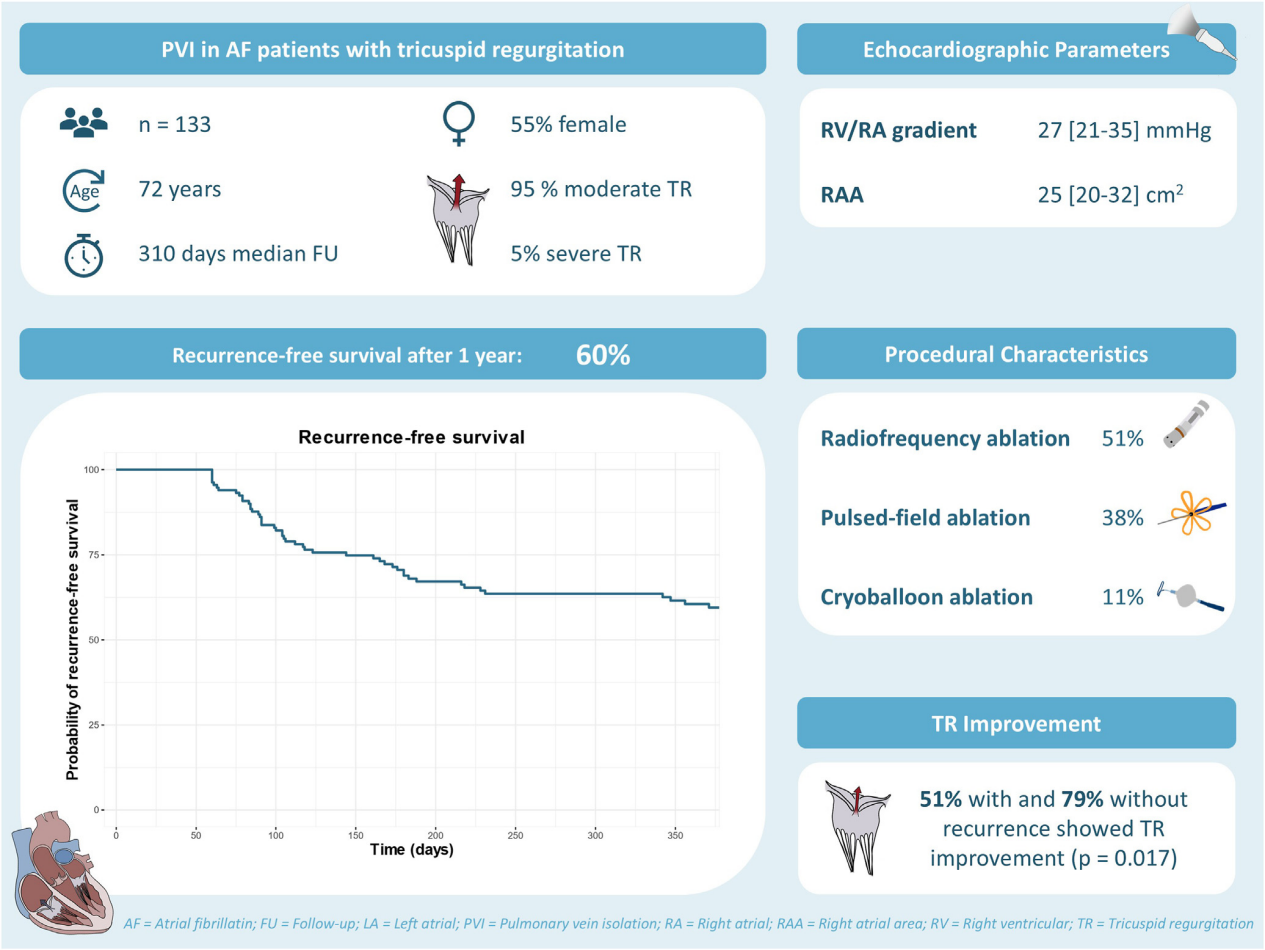
Impact of coexisting tricuspid regurgitation (TR) on procedural characteristics and the efficacy of pulmonary vein isolation (PVI) for AF.

The median procedural duration was 68 minutes (54–84 minutes), with a left atrial dwell time of 49 minutes (36–63 minutes) and a fluoroscopy time of 8 minutes (4–13 minutes). Additional ablation lesions were performed in 47 patients (35%), with posterior wall isolation being the most common (24 patients, 51%). Two complications (2%) were observed: 1 case of pericardial tamponade requiring drainage and 1 case of sinus arrest necessitating pacemaker implantation 1 day after the procedure. The median follow-up time was 310 days (105–526 days), and the 1-year Kaplan-Meier estimate for atrial arrhythmia recurrence-free survival was 60% (Figure 1). Follow-up ECGs were performed after a median of 230 days (121–414 days) after CA. Through follow-up, 63% of patients showed an improvement in TR severity by at least 1 grade after CA. When comparing TR improvement between patients with or without atrial arrhythmia recurrence, a higher proportion of patients without recurrence showed TR improvement (51% vs 79%, *P* = .017). There was no significant difference between CA modalities. Among the 133 patients undergoing CA for AF with moderate or severe TR, 2 patients (2%) with severe TR underwent interventional tricuspid valve reconstruction during follow-up.

The findings of this study corroborate and extend previous research on the efficacy and outcomes of CA in patients with AF and valvular heart disease.^1^^,^^2^ Our findings extend what has been demonstrated for the mitral valve to the often-overlooked tricuspid valve. The observed improvement in TR in 63% of patients during follow-up after CA is consistent with findings from 2 prior pilot studies.^3^^,^^4^ Importantly, patients without arrhythmia recurrence demonstrated significantly greater TR improvement, suggesting a possible induction of positive reverse atrial and tricuspid annular remodeling. These results underscore the role of achieving durable rhythm control in this challenging patient population to optimize clinical outcomes. The 1-year recurrence-free survival rate in patients with moderate to severe TR appears to be lower compared with rates reported in a general AF study population.^5^ Additionally, this is the first study including patients with moderate or severe TR undergoing pulmonary vein isolation using pulsed-field ablation. Limitations include the single-center design, the lack of a control group receiving anti-arrhythmic therapy, and the lack of a standardized interventional protocol for patients with persistent moderate or severe TR after CA for AF.

In conclusion, this study highlights the importance of durable rhythm control in patients with coexisting TR undergoing CA for AF. Further studies are warranted to confirm these findings and to explore optimal management strategies for patients with persistent TR following CA.
